# The Role of Cultural Beliefs, Norms, and Practices in Nigerian Women’s Experiences of Sexual Abuse and Violence

**DOI:** 10.1177/10778012211000134

**Published:** 2021-04-05

**Authors:** Chinyere Elsie Ajayi, Khatidja Chantler, Lorraine Radford

**Affiliations:** 1 University of Central Lancashire, Preston, UK; 2 Manchester Metropolitan University, UK

**Keywords:** cultural beliefs, norms, and practices, sexual abuse and violence, Nigerian women, feminist theory, intersectionality

## Abstract

This study aims to explore if and how cultural beliefs, norms, and practices might contribute to Nigerian women’s experiences of sexual abuse and violence. In-depth narrative interviews were conducted with 12 women of Nigerian origin living in the Northwest of England who had experienced sexual abuse and violence. Women’s accounts were analyzed thematically, and drawing upon a feminist-intersectional conceptual framework, analysis reveals that male privilege defined by gendered role and expectation, religious beliefs, rape myths, and bride-price with the associated practice of libation may have contributed to women’s experiences of sexual abuse and violence.

## Introduction

The Office for National Statistics (2014) reports that England and Wales have become more ethnically diverse, with rising numbers of people identifying as belonging to minority ethnic groups in 2011 compared with the previous three decades. This increase in the Black and minority ethnic (BME) populations implies cultural diversification that might mean new patterns of gender-based violence which are dissimilar to those experienced by the majority group (Smeaton, 2013). Examples of this include forced marriage and child marriages, female genital mutilation (FGM), and bride-price. To this end, there is a growing number of studies documenting the pattern, nature, and impacts of violence against women (VAW) of BME origin, including barriers to help-seeking and women’s experiences of support in the United Kingdom. However, these studies have largely focused on domestic VAW in South Asian communities (e.g., Ahmed et al., 2009; Anitha, 2008, 2019; Belur, 2008; Chantler et al., 2017; Gangoli et al., 2006; Gill, 2004; Gill & Harrison, 2019), thus, limiting the transferability of findings to other BME women in the United Kingdom. Some have considered South Asian women alongside other minority groups (e.g., Batsleer et al., 2002; Gangoli et al., 2020; Hester et al., 2007; Mama, 1989; Thiara & Gill, 2012; Thiara & Roy, 2010) and one, focused on African and Caribbean women (Kanyeredzi, 2018). Nigerian-born residents in England and Wales and the Nigerian community is recorded as one of the largest and most rapidly growing African communities in the United Kingdom (Communities and Local Government, 2009); yet, only one qualitative study (Femi-Ajao, 2018) specifically examined Nigerian women’s experiences of intimate partner abuse and violence in the United Kingdom. In consequence, very little is known about how Nigerian women experience gender-based violence, in particular, sexual abuse and violence. Similarly, there is minimal research on immigrant Nigerian women’s experiences of violence in diasporic countries. Existing studies (Kalunta-Crumpton, 2015, 2017; Nwosu, 2006; Ogbuagu, 2012; Ogunsiji et al., 2011) are all qualitative studies conducted in the United States, Canada, and Australia. While aligning with the assertion that cultural beliefs, norms, and practices may play a role in perpetuating VAW and violence against girls (Heise & Manji, 2016), the present study makes an important contribution to knowledge as it examines culture-specific factors that might contribute to Nigerian women’s experiences of sexual abuse and violence.

This article first draws on the aforementioned studies and Nigerian research to outline some of the potential contributory factors to BME women’s experiences of violence. It then situates the study within two interrelated theoretical frameworks, feminism and intersectionality. Next, the research method is described followed by the research findings. A discussion of the findings and implications for policy and practice concludes the article.

## Un–derstanding Factors Contributing to Violence Against BME Women

Most of the studies conducted in the United Kingdom report on a number of structural factors that influence BME women’s experiences of violence. These include poverty, poor socio-economic background and racism (Anitha, 2019; Gangoli et al., 2020; Kanyeredzi, 2018; Mama, 1989), insecure immigration status, and women’s level of acculturation (Anitha, 2008; Batsleer et al., 2002; Femi-Ajao, 2018; Gangoli et al., 2006, 2020; Gill, 2004; Thiara & Gill, 2012; Thiara & Roy, 2010). However, findings concerned with culture-specific factors in the United Kingdom are limited. Anitha (2019) found that gendered norms and hierarchy contributed to the exploitation of women’s productive and reproductive labor. Other studies found that religion was used to manipulate and trap women in abusive relationships. In addition, men used religion to assert gendered expectations on women such that when women resisted expectations of them as a “traditional wife,” violence was sometimes used to get them to conform (Gangoli et al., 2020; Mama, 1989; Thiara & Gill, 2012). With regard to disclosure and help-seeking, studies found that the desire to maintain their “honor” within their community, cultural norms acquired through socialization, and gendered power relations were key features in women’s narratives of their inability to seek help and escape abusive relationships (Ahmed et al., 2009; Femi-Ajao, 2018; Gill & Harrison, 2019). Similar findings were reported in a scoping review examining socio-cultural risk factors affecting domestic violence among South Asian immigrant women in English-speaking countries (Rai & Choi, 2018). Rai and Choi (2018) identified lack of social support, acculturation, enculturation, isolation, and patriarchal ideologies that support gendered expectations and male use of VAW as factors influencing women’s experiences of violence. Some of these findings are echoed within studies on violence against Nigerian women living in diasporic countries. Nwosu (2006) and Ogunsiji et al. (2011) found that structural factors such as racism, institutional discrimination, insecure immigration status, and unemployment affected men’s role as the breadwinners such that they sometimes resorted to the use of violence to assert their authority and superiority in the home. Cultural factors–for example, gendered expectations, male privilege, and boy-preference in childbirth–were also reported as contributing to women’s experiences of violence (Kalunta-Crumpton, 2015; Nwosu, 2006; Ogbuagu, 2012).

Qualitative studies conducted in Nigeria also suggest that Nigerian women’s experiences of violence have cultural underpinnings. Although feminist activism to address cultural beliefs, norms, and practices that might contribute to VAW in Nigeria, such as those organized by Female in Nigeria (FIN) on social media platforms, appear to have some influence in shifting views regarding norms and practices like rape myths, child marriage, and FGM (see Olofinlade, 2017), progress remains slow. It is argued that VAW in Nigeria is a socially accepted male behavior as well as a mechanism for curbing transgression against the culturally perceived male superiority (Abayomi & Olabode, 2013; Nelson, 2017). Studies examining gender ideology in promoting VAW in Nigeria have linked the belief in male superiority, grounded in the acceptance of gendered roles and expectations to discrimination against women and general tolerance of VAW (Fakunmoju et al., 2016; Odimegwu et al., 2010). Likewise, religious beliefs that subscribe to the idea that the man is the head of the family and has greater control and decision-making powers have been associated with VAW in Nigeria (Abayomi & Olabode, 2013). According to Amusan et al. (2017), patriarchal ideologies embedded in religious beliefs may advance the entrenchment of male dominance in social life, the oppression of women, and the excuse of men’s use of VAW in Nigeria.

Other studies (e.g., Odimegwu et al., 2010; Okenwa-Emegwa et al., 2016) examining women’s attitudes toward violence found that women themselves are sometimes accepting of men’s use of VAW. This could be a risk factor as evidence suggests that permissive social norms are positively associated with women’s experiences of violence in an intimate relationship (Odimegwu et al., 2010; Okenwa-Emegwa et al., 2016). Violence in marriage stemming from the inequality resulting in the practice of bride-price, “a payment from the groom or groom’s family to the bride’s family at the time of marriage,” is another case in point (Jasinski, 2001; Lowes & Nunn, 2017, p. 117). According to Horne et al. (2013), this practice diminishes women’s sexual and reproductive autonomy in marriage; as a result, rape within marriage is not recognized. Furthermore, studies connect sexual violence in Nigeria to the acceptance of rape myths and the culture of silence about acts of rape (Achunike & Kitause, 2014; Peters & Olowa, 2010). As Awosusi and Ogundana (2015) assert, the acceptance of rape myths in Nigeria means that the reporting of rape can be counter-productive for women as they could become revictimized by the patriarchal Nigerian criminal justice system. It is known that extant laws on rape in Nigeria are subject to customary norms and practices which oftentimes are biased against women. Consequently, laws which are meant to ensure gender equality in representation and adjudication of cases may actually penalize women and/or place them at an additional disadvantage (Awosusi and Ogundana, 2015). On the contrary, there have been some enforcement benefits of recent laws prohibiting all forms of violence such as the Violence Against Persons Prohibition Act (VAPP) 2015. This law aligns with the Convention on the Elimination of All Forms of Discrimination Against Women (CEDAW), 1979, to which Nigeria is a signatory. Sexual harassment claims–such as that of Pastor (Mrs.) Abimbola Patricia Yakubu v. Financial Reporting Council of Nigeria & Anor; Stella Ayam Odey v. Ferdinand Daapah & Cuso International; and Dorothy Adaeze Awogu v. TFG Real Estate Limited–decided in favor of women by the National Industrial Court of Nigeria (NIC) have set the pace in advancements in sexual harassment cases (for a detailed discussion of the cases, see Peters, 2019).

## A Feminist-Intersectional Conceptual Framework

Findings from the few, mostly small qualitative studies reviewed give some support to the feminist theory for understanding VAW. Findings suggest that VAW of Nigerian origin is a consequence of systems of domination that structurally and interpersonally place women in a subordinate position to men, increasing their vulnerability to domestic abuse, sexual abuse, and violence. Feminist theory rests heavily on the concept of patriarchy (Dobash & Dobash, 1979) and the social institutions which help to maintain it (Jasinski, 2001). Hunnicutt (2009) defines patriarchy as “systems of male domination and female subordination” (p. 553). It can also be conceptualized as male power over women (Hartmann, 1979) and sexual hierarchy, embedded in the gendered roles of women in the society (Eisenstein, 1979). A feminist analysis of VAW conceptualizes violence as a critical component of the system of male power (Yllö, 2005), thus placing gender and power at the center of the explanatory framework for understanding VAW. It also permits a deeper understanding of how gender and power operate both structurally (women’s access to and positions within social institutions) and ideologically (the beliefs, norms, and values about the status and roles of women in a society) (Dobash & Dobash, 1979). It is therefore useful in studying women’s lived experiences of violence in societies with strong patriarchal ideologies (Kalunta-Crumpton, 2017). Patriarchy is not only known to support VAW but also gender inequalities and disadvantages (Brownmiller, 1975; Yllö & Bograd, 1988). In Nigeria, for example, figures from British Council (2012) indicate that the existing patriarchal structure in Nigeria restricts women’s roles such that women occupy fewer than 30% of all posts in the public sector and only 17% of senior positions, and regardless of their educational qualifications, women were found to earn consistently less than their male counterparts. Practices like child marriage and bride-price are also known to promote gender inequality (Asagba, 2014; Uwe et al., 2007). Such practices violate the rights of girls to education and affords them very little opportunity in life, thereby continuing the feminization of poverty and male dominance in the society (Otoo-Oyortey & Pobi, 2003).

Studies reviewed also show that other additional factors inherent in women’s new environment influenced their experiences of violence. This included structural factors such as poverty, racism, insecure immigration, and economic-based gendered role reversal (Femi-Ajao, 2018; Nwosu, 2006; Ogunsiji et al., 2011). Therefore, the concept of intersectionality will also be applied in this study. Crenshaw (1989) introduced the term “intersectionality” to address the fact that the experiences and struggles of women of color did not receive enough attention by feminist theory or by anti-racist politics (p. 153). She asserts that feminist theory does not speak to the experiences of women of color; instead, the experiences of middle-class, educated, White women are used to implicitly reflect those of Black women. In the same vein, Crenshaw (1989) critiqued anti-racist politics for concealing Black women’s experiences of racism and sexism under that experienced by Black men. She argued that both feminist theory and anti-racist discourse addressed Black women’s experiences using frameworks that recognized gender and race as separate systems of oppression. Consequently, only issues raised by either sexism or racism were tackled, but not issues raised by the intersection of the two. This, she claims, provided a partial and distorted view of the experiences of Black women. She argues that “because the intersectional experience is greater than the sum of racism and sexism, any analysis that does not take intersectionality into account cannot sufficiently address the particular way black women are subordinated” (Crenshaw, 1989, p. 140).

## Research Methods

This study was approved by the PSYSOC Research Ethics Committee of the University of Central Lancashire. The aim was to recruit Nigerian women living in the United Kingdom who had experienced sexual abuse and violence. Therefore, to access women who met the inclusion criteria, groups or organizations where Nigerian women regularly attended were identified. Five organizations that work directly with BME women, including refugees and asylum seekers, were contacted via emails and telephone calls, and three responded positively. Working via gatekeepers, potential participants were provided with written information about the study; the researcher also spoke to women in groups about the study. Purposive and snowball sampling were also used to recruit participants (Curtis et al., 2000). A total of 12 women were recruited: 10 through organizations that work directly with BME women including refugees and asylum seekers, and two recruited through the researcher’s direct contacts.

Informed consent was renegotiated before the commencement of the interviews and obtained via a consent form signed by both the participant and researcher. Study participants were interviewed in English and all interviews were conducted between May 2017 and August 2017. This study utilized the “telling of stories” through conducting in-depth narrative interviews (Clandinin & Caine, 2008). An interview guide was used in all interviews and contained details of background questions for building rapport and possible follow-up questions to help elicit further and richer narrations from participants. Interviews were conducted in settings that participants chose and lasted between 30 and 60 min. Interviews were audio-recorded, and each participant received either £10 cash or a £10 gift voucher as a “thank you” for sharing their story.

The ethics of safeguarding women who participated in this study were very important. The recruitment method meant that women were pre-warned about the sensitive nature of the research. The pace of the interview was dictated by participants and regular breaks were offered to participants; however, none took up the offer. Follow-up questions were asked in a supportive, nonjudgmental manner and were carefully phrased to ensure participants had control of how much information they wished to offer. In addition, provisions were made to stop the interview in case of participant distress. According to Parker and Ulrich (1990), ending the interview on a positive note is essential in interviewing vulnerable groups. Therefore, after every interview, women’s strength and courage were reaffirmed, in some cases, using specific examples from their narrative. In addition, participants were debriefed using a debrief sheet which contained information and contact details of three legitimate, relevant, and accessible sources of help in case they required further support.

## Data Analysis

The audio-recorded interviews were listened to three times: first, for familiarization with the data; second, for verbatim transcription of data; and third, to fill in gaps missed by the first transcription. Each transcript was assigned a pseudonym to protect participants’ confidentiality. Transcripts were uploaded to NVivo 11 qualitative data analysis software before applying Braun and Clarke’s (2006) six phases of thematic analysis. The question of returning transcripts to participants for checking was posed during data collection, and all participants declined to revisit their stories (Lincoln & Guba, 1985).

### Interview Participants

The participants for this study were 12 women of Nigerian origin living in the Northwest of England, aged between 27 and 46, who had experienced different forms of sexual abuse and violence. Only one woman was born in the United Kingdom, while for the rest, the length of residency in the United Kingdom ranged between 2.5 and 13 years. One woman had refugee status, eight were seeking asylum at the time of the study, and the remaining two were refused asylum seekers. Ten of the women were born in Nigeria and one was born in another West African country. Women experienced sexual abuse and violence perpetrated by a church minister (*n* = 1), a stepfather (*n* = 1), an unknown male (*n* = 1), intimate partners (*n* = 8), a family friend (*n* = 1), and an auntie’s husband (*n* = 1). All the perpetrators are of Nigerian origin and are adult males, except in two cases of female genital mutilation (FGM), where parents (male and female) were named as perpetrators (*n* = 2). All the women in this study reported different types of sexual abuse and violence perpetrated both in Nigeria and the United Kingdom. These included childhood sexual abuse (CSA) = 2, sexual assault = 2, rape = 2, sex trafficking = 1, intimate partner sexual abuse and violence [IPSA/V] = 8, and FGM = 2. The names used in this study are pseudonyms to protect the women’s identity.

## Cultural Beliefs, Norms, and Practices

Four themes were identified in women’s narratives as factors which might have contributed to their experiences of sexual abuse and violence. These are: (a) male privilege defined by gendered roles and expectations, (b) religious beliefs, (c) rape myths, and (d) bride-price and the associated practice of libation.

### Male Privilege Defined by Gendered Roles and Expectations

Two women spoke about the privilege ascribed to men in Nigeria and how it is engraved in the Nigerian society through socialization: “If not that I came to the UK, my dear, I must tell you the truth, we are brought up like that, the man has the final say, he is the head of the house” (Asaro). Another commented,

em because of our upbringing, we think the man is the head of the house, he has the final say, so all you need to do is yes Sir, yes Sir, even when he is wrong you should be going yes Sir. (Sarah)

“Brought up” and “upbringing” suggest a socialization process, and as noted by Boudet et al. (2013), it is a key factor in the acceptance of gender inequality. These extracts also show that exposure to alternative discourses in a new environment may help shift learned and internalized gendered beliefs (Marcus & Harper, 2014). Because of the perceived superiority of the male, there is an expectation that married women behave in a manner that is respectful and submissive toward their husbands. These expectations were mostly communicated by the woman’s family and the partner. One woman described how her family condoned the sexual abuse and violence she suffered because of the perceived need to submit to her husband. She said,

I was being told that because of tradition I need to obey my husband, whatever he asked me to do even if am comfortable doing it or not, I need to do it. I couldn’t argue because it is old days tradition and they will tell you if you don’t do this, you are not respectful, you are not a good wife, you are not this . . . they will give you all kinds of names. (Sarah)

This extract illustrates how the interaction of gender and gendered roles shape and determine power dynamics in intimate relationships. A further justification for sexual abuse and violence was the need to fulfill the gendered and marital obligation of bearing children as observed in Sarah’s and Asaro’s extracts, neither of whom had any children at the time of the study.

In a place where the husband is drunk, he will just come home just start to struggle with the woman and try to have sex with the woman but in this case the woman cannot say anything and you cannot report the man to any of the family members because they will tell you, he got married to you because he wanted children, when he wants to have sex with you, he will have it at any time. (Sarah)

Asaro also stated,

Even when you are not comfortable having like sex or whatever, he will force you to do it because, he will say, I got married to you because you need to have children. I have to have sex with you anytime I want, you are my property, which now that am here, I find it that it is really abusive, but when we are back home, we took it that, that is what tradition expects us to do.

It appears that the collective acceptance of gendered roles by both the man and the woman’s family suggest that gendered roles are not to be contested by women. Those who deviate from these perceived norms are viewed less favorably by their immediate community; thus, women are left with no option but to remain in abusive situations.

### Religious Beliefs

Religion was found to be an important part of the participants’ lives with regard to coping with their experiences of sexual abuse and violence. However, some spoke of religious beliefs as a system that reinforced the notion of male superiority, as this extract from Asaro illustrates: “He will always say, he is the man, he is in charge, that kind of . . . I should always obey him, and he keep reminding me that the Bible says woman be submissive.” Religious beliefs also shaped unequal gender attitudes and social interaction, as observed when Lola reported on how her friends’ religious beliefs influenced their advice to remain in the abusive relationship. She said,

My Christian friends told me to pray. My Muslim friends told me the same thing. He is your husband, and they were saying it with all the love and all the care, it is not that they meant harm, they didn’t want to harm me. What they were doing was pushing me deeper into this circle of violence. (Lola)

The phrase, “he is your husband,” as used in the extract seems to emphasize the relationship between religiosity and commitment to marriage (Mahoney et al., 2001), which could be viewed in two ways. First, religious affiliations are key in determining matters regarding marriage and divorce (Casanova & Phillips, 2009) and second, divorce within a religious context contradicts the cultural norm prescribed for women (Seguino, 2010). Their suggestion “to pray” indicates that Lola’s friends struggled with the conflict of advocating for her to remain in the relationship and their disapproval of the abuse; nonetheless, their intervention embodied norms and stereotypes based on patriarchal religious beliefs which prescribe passivity and compliance. Religion was also used to minimize sexual abuse and violence. Angela, who was sexually abused by her stepfather, applied her Christian belief in making sense of the perpetrator’s actions as this extract shows:

I don’t know what was going through his mind at the time. Am a Christian as well, so you know, I could say that, and a lot of Christians can say that it was the devil that was putting things in his mind and making him behave the way he was behaving because generally, he has got a really good heart and he is a lovely man. (Angela)

Because religious beliefs shape everyday behaviors and decision making (Seguino, 2010) as this extract indicates, it is possible that when sexual abuse and violence are viewed through a religious lens, it may undermine the seriousness of the offense. It may also lead to the separation of the abuser from the abuse, which may hinder the victim from taking actions to prosecute the offender or even seek help.

### Rape Myths

Rape myths are major factors justifying sexual VAW in every society (Vonderhaar & Carmody, 2015). Lonsway and Fitzgerald (1994) define rape myths as “attitudes and beliefs that are generally false but are widely and persistently held, serve to deny and justify male sexual aggression against women” (p. 134). Two women’s accounts illustrate how rape myths could be used to minimize the severity of sexual abuse and violence. The following extract describes Efe’s experience of rape and her brother’s response.

. . . I said armed robbers just came to the house now, they (brothers) said where are they, where are they? I said they just raped me. I started crying and then he (brother) holds me. After, we now sat down together and everything, he (brother) said, I want to tell you something, this thing that happened to you, don’t say it out to anybody. You know when you say it out, they are going to use it to laugh at you. I said ok. I kept it. (Efe)

In the face of rape myths embedded in a culture of male dominance and the silencing of women’s voices, it is perhaps not surprising that Efe was silenced by her brother. It goes further to show how patriarchal societies such as Nigeria could be unsympathetic toward victims of rape. Indeed, it is possible that Efe’s brother was trying to protect her from the social stigma that would follow if she disclosed the rape. This exemplifies how rape myths can be subtle (McMahon & Farmer, 2011) and further indicates that rape myths not only function at a societal level but also at an interpersonal level, as prescribed beliefs shared by individuals (Edwards et al., 2011).

Angela, who was sexually abused by her stepfather in the United Kingdom, speaks of how her mother employed rape myths in responding to the sexual abuse she experienced: “. . . she (mother) would just say to me, make sure you lock your door before you go to bed and things like that.” It seems that Angela’s mother placed the responsibility for protecting herself from being sexually violated on Angela, who was a child at the time of the abuse. This could be seen to align with the rape myth that suggests that a woman deserves to be sexually violated if she does not take precautions to protect herself from the perpetrator (Xenos & Smith, 2001). This observation is consistent with the view that those who hold beliefs regarding the expected roles of women may also tend to accept rape myths (Burt, 1980); thus, when gendered roles and expectations come into play, the perpetrator is exonerated.

Although it seems that Angela’s mother colluded with the perpetrator (Bernard, 2001), it may be, as Brownmiller (1975) argues, that sexual violence is perpetuated by a patriarchal system where men hold higher status and have greater power than women, as this extract from Angela further illustrates:

there was a lot of arguments, he (stepfather) was trying to be authoritative . . . so she (mother) used to say to me, I must call him dad, I said no he is not, so she used to allow him to slap me and things like that, you know.

Angela’s mother asserted beliefs associated with gendered expectations through her response, as Angela states, “and my mum used to say things to me like, make sure you wear bra, don’t wear shorts, you know little little things like that.” This extract aligns with the rape myth that suggests that the woman asked for it through her provocative behavior or revealing dress (World Bank Group, 2019). Thus, her mother’s response signifies a clear socialization in the belief that women and girls can minimize their risk of rape or sexual abuse and violence by modifying their behaviors, including dressing in a socially acceptable manner (Vonderhaar & Carmody, 2015).

### Bride-Price and the Associated Practice of Libation

Bride-price is a practice that is widely observed in many regions of the world, including Southeast Asia and sub-Saharan Africa (Anderson, 2007). Bride-price has been reported to “bond families together, serve as an appreciation for the ‘gift’ of the wife to the husband, and in many cases women are ascribed more value and status in their marriages because of the bride price paid for them” (Hague et al., 2011, p. 556). This view was reflected in one woman’s account, as she provides a background to her narrative of sexual abuse and violence experienced in the United Kingdom.

Aunty said go and do it properly so that the man’s family will respect you as well. Let’s say in Nigeria, especially in Nigeria, if you marry, they have to marry you properly. If not, you don’t have respect, nobody will respect you. (Omola)

Although this practice may command some respect for women, on the contrary, some women believed that their experiences of sexual abuse and violence were influenced by the practice of bride-price. Asaro, who did not have children at the time of the study, described how her mother responded to her experiences of sexual abuse and violence at the hands of her ex-husband.

. . . my mother was like, no you have to stay with him, after all you are married, you are married. He has paid your bride-price just stay and endure, with time he will change, talk to him he will change. (Asaro)

Asaro’s mother’s response influenced Asaro’s decision to remain in the abusive relationship longer. Although women were aware that the bride-price could be returned, some spoke of the power dynamics inherent in the family structure that acted as a barrier. Women explained that in practice, family members are reluctant to return the bride-price due to the stigma attached to single parenthood and, possibly, the inability to bear the financial burden associated with returning the bride-price (Mazibuko, 2016; Onyango, 2016). Temi speaks of how she was encouraged to exercise continual perseverance to avoid the stigma of single parenthood: “Everything that he (ex-husband) is doing, my mother will tell me that I should endure, I should endure. I should do this, I should do that, do you want to become a single parent?.” She further states,

. . . you know back in Nigeria the stigma of not being in husband’s house . . . people will be mocking you, even your parents and it will be another ball game, that you cannot stay in your husband’s house? Even when they are killing you, they believe that you have to stay there. (Temi)

In conceptualizing the stigma and shame of “not being in husband’s house,” it is possible to see the interactions of gender, gendered expectations, and the practice of bride-price influencing the accommodation of violence in marriage by both Asaro’s and Temi’s mothers. Both Asaro and Temi understood that their families would disapprove of them leaving the marriage; thus, they remained in abusive relationships for fear of being seen as not conforming to gendered expectations (World Bank Group, 2019). One woman further associated the practice of bride-price to a ritual practice of pouring out a drink as an offering to a deity, also known as libation. Her account suggests that the practices of bride-price and libation happen concurrently, which works to trap the woman in the marriage for fear of repercussions associated with the ritual practice. Omono illustrates how this practice might be used to instill fear in women, thus keeping them in a subordinate and powerless position. Even after separating from her husband for over two years, she claims that she cannot be involved with another man. She stated,

. . . they call it “Eriwe”—google it in the Urobo language. When they pour that libation and all those stuffs, no man can climb you. As I speak right now, no man can climb me. That is our culture, you can’t, when they do that libation and all those stuffs, if you dare, the man dies, or you die, or the baby in your womb will not come out. If you get pregnant from another man, your baby can stay there for three years, not until you will now make a confession and the person you make that confession to is your husband’s family members. (Omono)

This extract illustrates how gender, bride-price, and the practice of libation intersect to provide the context for disempowering women. Arguably, both inter-related practices potentially diminish women’s autonomy by suggesting that there are no alternatives to the abusive marriage.

## Discussion and Conclusion

The aim of this study was to examine if and how cultural beliefs, norms, and practices might contribute to Nigerian women’s experiences of sexual abuse and violence. Based on the narratives of 12 women of Nigerian origin who have experienced sexual abuse and violence, four key themes were identified and analyzed. These included male privilege defined by gendered roles and expectations, religious beliefs, rape myths, and bride-price and the associated practice of libation. These themes are linked and embedded within structures of patriarchy, which, in turn, gave power to systems that shaped the perpetration of sexual abuse and VAW. Findings also show that the cultural beliefs, norms, and practices identified in women’s narratives intersected with gender and systems of domination to influence women’s experiences of sexual abuse and violence.

### Male Privilege Defined by Gendered Roles and Expectations

Beliefs around the superiority of the man in the household are tied to his role as the breadwinner and the patriarch who makes all decisions (World Bank Group, 2019); so, by default, the woman occupies an inferior position defined by her role. As Uwe et al. (2007) argue, the way women are perceived in Nigeria is based on cultural norms and gendered expectations. The findings of this study align with this assertion and demonstrate how beliefs in gendered conditions differently position women and men and maintain relations of domination and subordination. Indeed, such gendered hierarchies are foundations on which gender inequality, oppression, and power are maintained. Furthermore, findings show that when sexual abuse and violence is viewed through the lens of gendered expectations, it could be interpreted differently. It is this type of wider interpretation that serves to justify sexual abuse and violence (Adames & Campbell, 2005; Krahé et al., 2005), thereby creating grounds for the continuation and the operationalization of subordination and powerlessness in women’s lived experiences. As seen in the findings of this study, the obligation to bear children completely diminished women’s sexual autonomy and reproductive rights within the marriage in a way that justified the perpetration of sexual abuse and violence. This finding strengthens the findings of quantitative studies like that of Iliyasu et al. (2016), which report a high prevalence rate of VAW attending infertility clinics in Nigeria. While sexual abuse and violence within marriage–that is, marital rape–also happens in the West, it appears that based on the findings from Iliyasu et al.’s (2016) study as well as the current study, Nigerian women who are unable to conceive may be vulnerable to sexual abuse and violence as a result of the culturally perceived marital obligation of bearing children for their husbands.

Findings also indicate that due to the expectation to adhere to cultural master narratives around gendered roles, conveyed through socialization, some women had little choice but to capitulate and tolerate their experiences of sexual abuse and violence. Therefore, this study proposes the concept of “family and community betrayal” in understanding women’s fear of the possible social ostracism that might occur if they left the abusive relationship. The concept of “family and community betrayal” is similar to the concept of “family honor” articulated in South Asian women’s experiences of violence (Gangoli et al., 2006; Izzidien, 2008). Such cultural master narratives around gendered roles and expectations also suggest that women are expected to be self-sacrificing to maintain the patriarchal structure. Unsurprisingly, feminist theoretical perspectives posit that this form of gender inequality will help predict the likelihood of VAW or create fundamental justifications for sexual abuse and violence (Dobash & Dobash, 1979).

### Religious Beliefs

Religion is an integral part of the lives of Nigerians and has been argued to support the overall well-being of women (Bernard, 2016; Para-Mallam, 2006). Inglehart and Norris (2003) note that although religious institutions are not monolithic, they still shape cultural norms, social rules, and behaviors that affect gender roles and expectations. Evidence from this study shows how the religious belief of wifely submission was reflected through religious interpretations that suggest that refusing sex is a form of deviance on the part of the woman. Feminists have argued that such beliefs embedded in the teachings of wifely submission are contributing factors to VAW (Dobash & Dobash, 1979) and, as Igbelina-Igbokwe (2013) contends, they have become “a critical weapon to enforce subordination” (p. 6). Furthermore, because such religious ideologies contribute to gender inequality and the perpetration of VAW, it is likely that women, who constitute most of religious memberships (Olajubu, 2008), will become less egalitarian. The fundamental influence of such attitudes was seen in one woman’s account of when her friends encouraged her to remain passive and compliant. This attitude could make it difficult for women to enact resistance, thereby leaving them vulnerable to further abuse (Levitt & Ware, 2006). Another example seen in this study is the tendency to minimize sexual abuse and violence when viewing it through a religious lens. This informal impact of religion could also be far-reaching as it may potentially foster grounds for a systematic reproduction of women’s disadvantage and the sustenance of VAW in Nigeria and diasporic countries.

### Rape Myths

Arguably, rape myths serve largely to keep patriarchal ideologies and structures in place as they not only protect men from being held accountable for rape but also may justify the perpetration of sexual abuse and violence (Carmody & Washington, 2001). Evidence in this study supports the view that rape myths function at multiple levels. Thus, they not only have a profound effect by minimizing the severity of sexual abuse and violence but also work to further disempower women. Evidence further supports a relationship between rape myths and less favorable attitudes toward victims of rape (Boakye, 2009). It could be surmised, therefore, that the level of acceptance of rape myths in a society will determine the way the society responds to rape, which in turn will influence the level of disclosure of sexual abuse and violence. Rape myths also function through gender norms grounded in patriarchy which suggest that women and girls who wear certain clothes “invite” rape as it negates the culturally accepted behavior for women (Brinson, 1992). This is another way that gender and power intersect to limit women’s options with regard to sexual abuse and violence.

Although the role of rape myths in justifying sexual VAW in every society has been highlighted in the literature (Carmody & Washington, 2001; Vonderhaar & Carmody, 2015), an important finding of this study is that rape myths can apply across the life course. Rape myths linked to CSA that the female child is responsible for protecting herself from a sexually abusive stepfather by not dressing provocatively and by keeping the door of her bedroom locked illustrate how rape myths are used to place the responsibility on women and girls to prevent sexual abuse and violence. Findings further reveal how the intersection of cultural factors and gendered power relations in the home could leave mothers in an ambivalent position with no option but to intervene in their daughter’s experiences of intrafamilial sexual abuse using rape myths. While not supporting the use of rape myths in intervening in intrafamilial CSA cases, the role of mothers must be understood considering the culturally determined hierarchical family structures that leave women at the bottom, including factors such as the stigmatization associated with single parenthood. Also, based on a feminist perspective, the argument is that it is the interrelationship between gender and power that gives rise to the passive acceptance of rape myths by mothers. However, this form of passivity possibly indicates an implicit acceptance of gendered roles and expectations, thus breeding a culture whereby incidents of sexual VAW and sexual violence against girls are reframed as an act of deviance by the girl or the woman. This implies that although women in every society can be affected by rape myths, they are likely to be heightened in more patriarchal societies. Based on this view, the present study argues that rape myths are sustained by unequal gender relations that support patriarchal structures, interpersonal violence, and the subordination of women (McPhail, 2015). Therefore, with regard to transforming social attitudes toward rape myths among Nigerians and, indeed, in other societies, the relationship between gender and power–in other words, patriarchy–should become the focus of campaigns and research.

### Bride-Price and the Associated Practice of Libation

Despite some evidence pointing to some benefits of bride-price (Hague et al., 2011), findings from this study show that bride-price may increase male ownership of his wife’s body and increase the likelihood of sexual abuse and violence. Bride-price may also leave women who are experiencing different forms of abuse and violence in marriage with no recourse for escape, thus continuing the cycle of revictimization. The extracts from Asaro, Temi, and Omono suggest that, apart from their narratives being embedded within structures of patriarchy and heteronormativity, women were also faced with the complexities posed by the practice of bride-price. The collective cultural expectation to remain in a marriage after the bride-price is paid justified the stigma attached to a marriage breakdown and further reinforced women’s subordinate position in the family (Hague et al., 2011). These complexities interacted with gendered expectations at societal and family levels to further disempower women from acting, thus reinforcing their experiences of sexual abuse and violence. Kandiyoti (1987) describes this form of collective cultural expectation as a “corporate control over female sexualities . . . with extensive informal support systems” (p. 333). Findings also highlight how libation practices discriminate against women and disempower them from taking control of their lives. Because control was exerted on women’s autonomy regarding staying in abusive relationships through this ritual practice, it is possible to see how gender intersected with both libation and bride-price to form an overarching system of domination in women’s lived experiences (Collins, 1991). This is significant in our understanding of how beliefs associated with women’s country of origin may remain strong and play a crucial role in their lived experiences (Brettell, 2000).

## Implications for Policy and Practice

The findings of this study highlight the influence of cultural beliefs, norms, and practices in Nigerian women’s lived experiences of sexual abuse and violence and how the operations of intersectionality keep women in the abusive situation. Although tackling harmful gender norms and practices have been rightly highlighted in the refreshed “Ending Violence Against Women and Girls Strategy 2016-2020” document (Ending violence against women and girls 2016–2020 Strategy Refresh, 2019), there is a need to adequately accommodate inclusive and ethnic-specific measures in this strategy. This would mean a review of the measures so that it takes account of patriarchal cultural beliefs and norms that contribute to BME women and girl’s experiences of violence. Also, the needs of women with insecure immigration status who are subjected to harmful beliefs, norms, and practices need to be addressed so that such women are not further subjected to state policies that leave them with few or no alternatives.

The findings of this study are also relevant to service providers, particularly those supporting women to leave abusive relationships. It is imperative for services and professionals to recognize that the way Nigerian women or women from other BME backgrounds experience violence will be “qualitatively different from that of white women” (Crenshaw, 1991, p. 1245). Service providers also need to acknowledge the complexities arising from these cultural factors which may influence the effectiveness of intervention when working with BME women. This suggests that services and professionals will need knowledge of these cultural factors such that intervention strategies are responsive to the interaction of these factors to ensure holistic and meaningful support. This is central to culturally sensitive practice and avoiding the pitfalls of culture-blaming which may potentially exclude BME women from adequate support (Burman & Chantler, 2004). Future research could focus on comparing the perspectives of Nigerian men living in the United Kingdom and those living in Nigeria. Such research and analysis will help to build more insight and understanding into the issues of patriarchy; cultural beliefs, norms, and practices; and immigration, which would help to determine what further implications there might be regarding future policy and practice developments.

## Limitations of Study

The sample size of this study represents a very limited range of women from Nigeria living in the United Kingdom and could be seen as a limitation of this study. Also, due to the sensitive nature of this study, it was difficult to recruit target women from different settings, thus limiting the diversity of the women with regard to their immigration status. Nonetheless, because this study was conducted using a narrative method of inquiry, utilizing the “telling of stories” as a data gathering strategy (Clandinin & Caine, 2008), rich data were generated from the 12 participants which provided in-depth insight into women’s experiences of sexual abuse and violence and improved the authenticity and credibility of the study (Lincoln & Guba, 1985).
